# Consumption of Eggs Alone or Enriched with Annatto (*Bixa orellana* L.) Does Not Increase Cardiovascular Risk in Healthy Adults—A Randomized Clinical Trial, the Eggant Study

**DOI:** 10.3390/nu15020369

**Published:** 2023-01-11

**Authors:** Yeisson Galvis, Keilly Pineda, Juliana Zapata, Juan Aristizabal, Alejandro Estrada, María Luz Fernandez, Jacqueline Barona-Acevedo

**Affiliations:** 1 Research Group of Toxinology, Food and Therapeutic Alternatives, Universidad de Antioquia UdeA, Medellín 050010, Colombia; 2 School of Microbiology, Universidad de Antioquia UdeA, Medellín 050010, Colombia; 3 School of Nutrition and Dietetics, Universidad de Antioquia UdeA, Medellín 050010, Colombia; 4 Physiology and Biochemistry Research Group-PHYSIS, Universidad de Antioquia UdeA, Medellín 050010, Colombia; 5 School of Nutritional Sciences and Wellness, University of Arizona, Tucson, AZ 85721, USA

**Keywords:** eggs, annatto, *Bixa orellana*, carotenoids, lipoproteins, apolipoproteins, blood lipids, lipid biomarkers, cardiovascular risk

## Abstract

Most atherosclerotic cardiovascular diseases can be prevented by modifying lifestyles, including unhealthy diets. Eggs contain important carotenoids that may impact cardiovascular risk. The lipid nature of eggs can improve the bioavailability of other carotenoids, such as Annatto (*Bixa orellana* L.), with reported antioxidant properties. Although numerous studies have shown that there is no association between egg consumption and cardiovascular risk, there is still controversy. In addition, there is limited information about Annatto’s effects on human health. This study evaluated the association between egg consumption and its enrichment with Annatto in lipid biomarkers of cardiovascular disease. In a parallel clinical trial, one hundred and five (*n* = 105) men and women were randomized by age, sex, and body mass index (BMI), and distributed into three groups. Subjects consumed daily, for 8 weeks, either two eggs, two eggs with Annatto, or two egg whites. Plasma lipids were measured by enzymatic colorimetric methods, plasma apolipoproteins and lipoprotein subfractions and size by nuclear magnetic resonance. There were no differences between groups in age, sex, and BMI. No significant changes were found over time or between groups in plasma triglycerides, LDL cholesterol, HDL cholesterol, apolipoprotein (apo) A1, apo B, or lipoprotein subfraction concentrations. In healthy adults, the intake of two eggs a day, or two eggs with Annatto for eight weeks, did not generate adverse changes in cardiovascular risk markers.

## 1. Introduction

Most atherosclerotic cardiovascular diseases (CVD) can be prevented by acting on behavioral risk factors, such as unhealthy diets and physical inactivity [1]. Furthermore, the risk of suffering from these diseases grows with the increase in obesity [2]. According to the nutritional situation survey in Colombia (ENSIN, 2015), excess body weight (overweight or obesity) in adults (18–64 years) increased by 5.2% from 2010 to 2015 [3,4].

Colombia is a country with medium to low incomes and whose poverty has increased with the pandemic [5]. Thus, the economic capacity of Colombians to acquire a balanced diet, which includes quality protein, abundant fruits and vegetables, and healthy fats, is reduced. Egg protein is considered the most bioavailable and complete, among the quality proteins. It is the standard of comparison for other protein sources, with the advantage of being one of the cheapest animal protein sources with an excellent content of essential amino acids, providing a moderate caloric content [6].

For nearly 50 years, dietary cholesterol and eggs were thought to contribute to increased plasma cholesterol and CVD risk [7]. In 1968, the American Heart Association recommended consuming less than 300 mg/day of dietary cholesterol and no more than three eggs per week. These restrictions affected not only the egg industry, but also poor communities, who labeled the egg as a high-cholesterol food and reduced their consumption of this low-cost and nutritious food [8].

Follow-up studies have shown that egg consumption has been associated with a lower mortality risk [9], with the formation of larger and less atherogenic low-density lipoprotein (LDL) and an increase in high-density lipoprotein (HDL) cholesterol, improving lipid metabolism [10]. However, there is still controversy in longitudinal studies that only evaluate classic markers such as total cholesterol, triglycerides, HDL-C, and LDL-C [11]. In these studies, egg consumption continues to be associated with the risk of mortality, persisting with the debate about egg consumption and cardiovascular risk.

In addition to protein and cholesterol, eggs also contain important carotenoids (antioxidants), such as lutein and zeaxanthin. Higher plasma concentration of these two carotenoids associated with higher concentrations of large LDL and HDL particles have been reported in people consuming three eggs/day for 12 weeks, compared to an egg substitute [12]. Moreover, the lipid nature of eggs can improve the bioavailability of other dietary carotenoids compared to different matrices or natural sources of carotenoids, such as raw vegetables [13]. Therefore, the ex vivo enrichment of eggs with a source of carotenoids could represent an alternative to increasing the plasmatic levels of these antioxidants in the population.

Carotenoids, the natural pigments of fruits and vegetables [14], have been evaluated for their antioxidant properties, but with controversial [15] or inconclusive [16] results. A source of carotenoids that has gained importance in recent years is Annatto (*Bixa orellana* L.), which, in addition to being used to provide color to foods, has been reported to have healing, anti-inflammatory, antioxidant, and antimicrobial properties [17,18,19]. However, some review articles suggest that more in vivo studies are needed to evaluate the nutrients and healing properties of Annatto [20], especially in Colombia and Latin America, where the production of Annatto is high [21,22], providing an alternative investment opportunity.

Given that the controlled evaluation of the effects of egg consumption and its enrichment with carotenoids (from Annatto) on cardiovascular health in the Colombian population is very limited, the objective of this study was to evaluate the association between egg consumption and eggs enriched with Annatto (*Bixa orellana* L.), and some biomarkers of cardiovascular risk, in an adult population.

## 2. Materials and Methods

This was a parallel randomized clinical trial with three intervention groups. One hundred and five (*n* = 105) men and women were distributed into 3 groups (*n* = 35 each), matched by age, sex, and body mass index (BMI). The sample size is based on a mean difference found in the change value in blood lutein (ng/mL) in a similar study [23]. Sample size was calculated using the Sample Size Program version 1.1. from the Pontificia Universidad Javeriana [24], with confidence of 95%, a ratio of 1 to 1, and power of 85%. The inclusion criteria were age 18 to 59 years; BMI between 18.5 and 29.9 kg/m^2^. Exclusion criteria included triglycerides >500 mg/dL, total cholesterol >240 mg/dL, plasma glucose >126 mg/dL or having been diagnosed with diabetes, blood pressure >140/90 mmHg, and suffering from or having had liver or kidney disease, cancer, endocrine disorders, heart disease or stroke, or intestinal disorders that limit nutrient absorption. In addition, the use of hypoglycemic and lipid-lowering medications and nutritional supplements (vitamins, minerals, fatty acids, etc.) was considered as an exclusion criterion. This clinical trial was registered on ClinicalTrials.gov, identifier: NCT05088577.

### 2.1. Intervention

**Figure 1:**
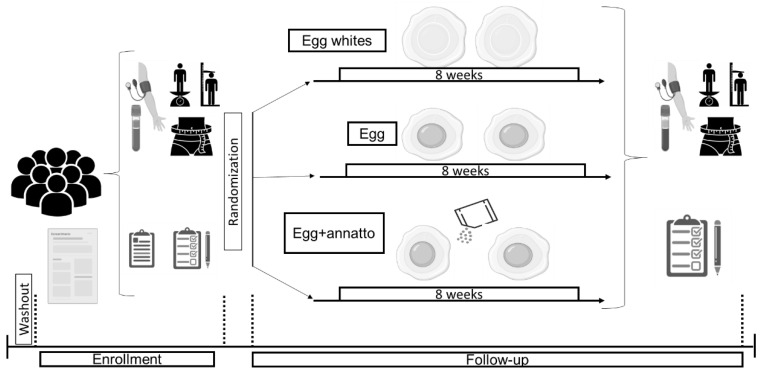
Study intervention protocol.

The recruitment of the participants took place between October 2019 and December 2020. The volunteers underwent an initial washout period of 2 weeks in which they were asked to not consume eggs. After signing the informed consent document, biochemical tests were done to ensure that no exclusion criteria were present. Subsequently, they were randomized by minimum using the ETCETERA statistical calculator of the WINPEPI program [25] to consume one of the following foods daily for eight weeks: 2 eggs, 2 eggs with Annatto, or 2 egg whites (control group). The eggs were provided by Avinal^®^ (Avícola Nacional S.A., Medellín, Colombia) from the same local farm. Pasteurized liquid egg whites were also provided by Avinal^®^, in a commercially available container. The dose of Annatto was the equivalent of 1.2 mg of bixin/kg of body weight for 2 eggs daily, a value that does not exceed that allowed for dietary addition [26] and that has been shown to have potential health effects [27]. Annatto powder (Badia^®^, Badia Spices, Inc., Doral, FL, USA) was bought from a local supermarket. Blood lipids and glucose, anthropometric measurements, and dietary assessments were obtained at the beginning and end of the intervention period. See Figure 1. Volunteers were asked to maintain their usual lifestyles during the study, except for the consumption of additional eggs, egg whites, or Annatto, respectively. Participants filled out a form each week to evaluate their adherence.

### 2.2. Blood Collection

Blood samples were collected from the antecubital vein using dried tubes after an overnight 12 h fast. After 30 min, the tubes were centrifuged at 450× *g* for 15 min, and serum was aliquoted and frozen at −70 °C for further analysis.

### 2.3. Anthropometric Measurements

Weight and height were measured using a stadiometer (SECA 216, seca S.A.S., Hamburg, Germany) and a scale (SECA 813, seca S.A.S., Hamburg, Germany). Then, BMI was calculated as kg/m^2^.

### 2.4. Blood Lipid Profile, Glucose, and Liver Enzymes

Total cholesterol (TC), HDL cholesterol (HDL-C), LDL cholesterol (LDL-C), triglycerides (TG), glucose, and liver enzymes (AST, aspartate aminotransferase; ALT, alanine aminotransferase) were measured via enzymatic colorimetric methods using an automatic analyzer (Siemens^®^, Erlangen, Germany) in a certified clinical laboratory facility.

### 2.5. Lipoproteins and Apolipoproteins

Nuclear magnetic resonance was used for the measurement of apolipoprotein (Apo) B, Apo A1 concentration, and the total number, size, and concentration of lipoprotein particles. The analysis was performed by Labcorp© (Laboratory Corporation of America Holdings, Burlington, NC, USA).

### 2.6. Diet Analysis

Diet was evaluated using a semiquantitative food frequency questionnaire (FFQ) at the beginning and last week of the intervention. Total energy (Kcal) and macronutrient intake (g) were estimated using a Colombian food database [28]. This FFQ has been validated in a similar population [29] and has been used in previous studies [30,31].

### 2.7. Statistical Analysis

A per-protocol analysis was performed with participants who demonstrated at least 80% adherence to the study. To describe the study population, a univariate analysis was performed with percentages and Pearson’s chi-squared test. For frequencies less than 5, Fisher’s Exact Test was applied. In addition, a group mean analysis was carried out using repeated-measures ANOVA (evaluating time, treatment, and time x treatment); the assumptions were verified, and when they were not met, transformations were carried out using the Box–Cox procedure. The analyses were done in the RStudio 4.0, 2021 program interface (Posit PBC, Boston, BSA, USA). *p*-values were estimated, establishing a level of statistical significance of *p* < 0.05. The graphs were built in the GraphPad Prism 5.0 program, 2021 (Insight Partners., New York, NY, USA). 

## 3. Results

**Figure 2:**
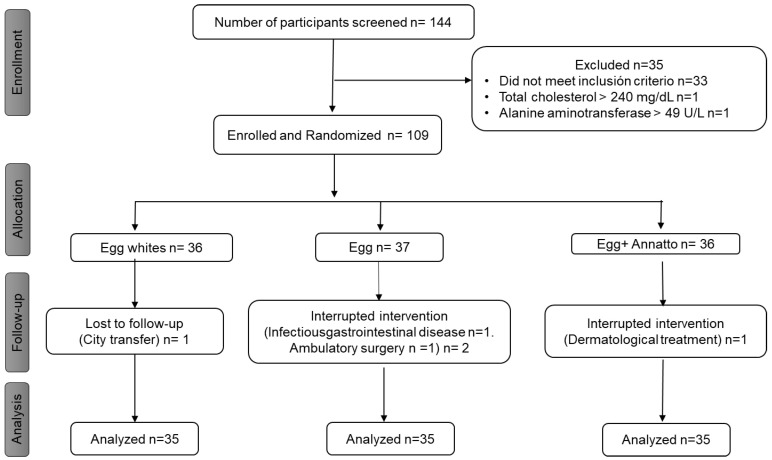
Study intervention flowchart.

One hundred and forty-four (*n* = 144) people were screened, 33 did not meet the inclusion criteria, and two more were excluded for having dyslipidemia and elevated liver enzymes, respectively. Four volunteers dropped out for reasons unrelated to the study (Figure 2). Finally, one hundred and five (*n* = 105) participants completed the study; 65.8% were women, with an average age of 28 years.

According to the randomization method, the proportion of women was the same in the three groups. Likewise, there were no differences between groups in age, BMI, and sociodemographic variables (Table 1). Regarding diet, there were no significant differences (*p* > 0.05) in nutrient intake between the groups in time or the interaction time x treatment.

The results obtained for the traditional cardiovascular risk variables, from blood lipid profiles and glucose, showed no significant changes either over time or by treatment; see Table 2. In addition, levels of liver enzymes, as markers of hepatic inflammation, were below the upper limit for AST (32 U/L) and ALT (49 U/L) at the end of the intervention for the whole group (AST: 23.8 ± 9.8 U/L; ALT: 28.3 ± 17.9 U/L). Moreover, there were no significant differences (*p* > 0.05) in the groups in time and the interaction time x treatment in these liver damage markers.

When Apo A1 and Apo B levels were obtained and the atherogenic indexes were calculated, no significant changes were found in any of the indexes or the Apo B and Apo A1 levels for the treatment groups in the two periods of time evaluated (Table 3).

When analyzing more advanced and specific markers of CV risk, no significant differences were found for any of the lipoproteins evaluated (*p* > 0.05). There were no substantial changes over time or in the treatments evaluated (Table 4).

**Figure 3:**
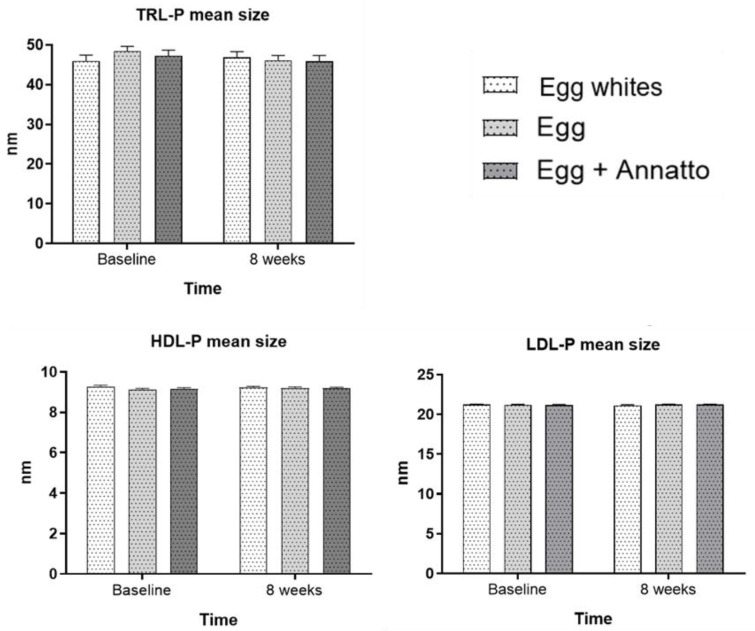
Mean triglyceride-rich lipoprotein particle (TRL-P), low-density lipoprotein particle (LDL-P), and high-density lipoprotein particle (HDL-P) sizes at baseline and after 8 weeks of consumption of egg whites, egg, or egg + Annatto. Repeated-measures ANOVA. Significance of treatment, time, and time x treatment *p* < 0.05 for all lipoprotein subclasses.

Additionally, the mean size of LDL, HDL, and triglyceride-rich lipoproteins was analyzed to evaluate whether significant differences were observed on average for each group (Figure 3). No significant results for any lipoprotein subclass were observed. It is important to note that in the egg white group, increases in the mean size for the triglyceride-rich lipoprotein (TRLP) subclass were observed (2.1%), as well as a slight reduction in the mean size for the egg (4.9%) and egg + Annatto groups (3.1%), respectively, for this lipoprotein subfraction.

## 4. Discussion

The main findings in this study were that, compared to egg whites, two eggs per day, alone or in combination with Annatto, resulted in similar concentrations of plasma LDL cholesterol, triglycerides, and apolipoprotein B, important biomarkers for cardiovascular disease risk [32]. Further, the number of atherogenic lipoproteins, including small LDL or large VLDL, was not different among groups, further emphasizing that two eggs per day do not unfavorably alter key biomarkers for CVD risk.

In this study, a higher percentage of women was recruited (Table 1), in contrast to the history in intervention studies evaluating lipids and cardiovascular risk, where women have had lower representation compared to men [33,34,35]. For other sociodemographic variables, there were no imbalances between the groups, indicating that the randomization process by minimums used in this study avoided the influence of these variables in the intervention and reduced possible biases.

Additionally, most volunteers (67%) were adults, with an average age of 28 years and with a medium income level (stratum 3). This specific population is important to evaluate, because, for countries such as Colombia, it represents the working class [36], where most people are concentrated, with medium to low income levels, and the double burden of malnutrition (undernutrition with obesity) is present [37,38]. Evidence shows that more than 50% of global deaths can be attributed to diet [39]. One hundred grams of edible egg contains 13 essential vitamins and minerals and high-quality protein (approximately 12.4 g), all for only 70 calories [6]. However, for nearly 50 years, eggs were evaluated based mainly on their cholesterol content and were thought to contribute to increased plasma cholesterol and CVD risk [7]. The findings of this study (seen in Table 2) show that the consumption of two eggs daily for 8 weeks did not increase cardiovascular risk measured with classical markers. These results agree with other studies showing that egg consumption is not related to increased risk [40,41,42,43] and contradict different results reported for other populations [11,44].

To contribute to the debate and present evidence against the causality between egg consumption and increased CV risk, it is necessary to go beyond classical biomarkers. Given that classic lipid biomarker profiles do not constitute a complete vision of the metabolic process, many authors base the risk assessment on atherogenic indices [45,46]. For this reason, it is important to evaluate the association between the consumption of eggs and the increase in CV risk through these indices in this study. From Table 3, it is evident that for all risk ratios evaluated, the indices did not change significantly (*p* > 0.05), neither over time nor by treatment, similar to the results reported in other intervention studies [41,47,48].

Some individuals with traditional lipid profile values at levels considered at no risk may have an atherogenic lipoprotein profile [49,50]. Therefore, it is necessary to explore more sensitive markers to assess CV risk. In Table 3, Apo B and Apo A1 and their related indices are evaluated. These markers allow an evaluation of CV risk based on the fact that apolipoproteins are structural components of lipoproteins and play a crucial role in the relationship between receptor binding and enzyme activation in the lipid metabolism process. In addition, Apo B is found in all atherogenic lipoproteins and Apo A1 in HDL (atheroprotective) lipoproteins. For this reason, the concentration of these apolipoproteins gives more specific information when evaluating CV risk [51,52,53]. There were no significant differences in Apo A1 and Apo B concentrations after egg consumption, versus the control group. The results clearly show no significant association between egg consumption and increased CV risk in a healthy population, evaluating either classic (lipid profile indices) or more sensitive biomarkers (apolipoproteins).

Atherogenic dyslipidemia is characterized by an increase in small and dense LDL particles, reduced levels of HDL-C, and increased triglycerides in plasma. Atherogenic dyslipidemia is more highly associated with CV disease (CVD) incidence than the presence of elevated LDL-C alone, indicating that it is a better predictor of CVD [54]. After evaluating the effects of egg consumption on these specific lipoproteins (Table 4), no significant changes were observed, comparable to the results reported by others evaluating similar populations and intervention studies [55,56]. Contrary results have been reported in studies where the control group did not consume egg whites or modifications to the participant’s diet were made [12,39]. In this study, participants were asked to maintain their usual diets, and not to include additional eggs or egg whites (control group) provided by the researchers. Some increases in lipoproteins have been reported [57,58]; however, the risk is not increased because significant changes have been observed for both large HDL and LDL particles, maintaining the proportion between atherogenic and protective lipoproteins.

Contrary to other intervention studies [23,27,42], no additional benefits were observed in the group in which carotenoids (Annatto) were added to the eggs; the results showed that there were no changes in the risk profile when consuming Annatto in the proportion supplied in the study. The Achiote plant (Annatto), rich in carotenoids (mainly bixin, but also norbixin and byproducts of lycopene), also contains important amounts of tocotrienols, tocopherols, terpenes, and flavonoids [59]. Given that no previous studies evaluating the effects of long-term supplementation with Annatto in humans were available in the literature, the dose used in this study was mainly based on the potential beneficial effects of this plant, reported in an acute study in healthy people [27]. A recent review article [60] recommends avoiding excess intake of single carotenoids >30 mg/d or more. A group of participants in this study consumed carotenoids from Annatto mixed with carotenoids of eggs (lutein and zeaxanthin); however, there were no signs of hepatic inflammation, given that the observed values of liver enzymes were in the normal range at the end of the intervention.

This study was exploratory for the lipid variables measured; thus, it is necessary to complement the evaluation with more specific variables, such as the increased carotenoids in participants’ blood and the antioxidant capacity, which might show more noticeable changes. However, this first study of long-term Annatto effects in a Colombian population motivates new research on the bioavailability and bioactivity of a product with great potential for Latin America.

The results observed in this study, in healthy middle-aged adults, after consuming two eggs per day for 8 weeks compared to egg whites, show no causal association, with no increase in CV risk as measured by a comprehensive panel of biomarkers, including both classical and more advance or sensitive ones. Several clinical studies have reported similar results to this study, demonstrating no association between egg consumption and increased risk for heart disease [56,58,61]; however, some studies continue the debate, reporting an association between egg consumption and CV risk [11]. Currently, the American College of Cardiology and the American Diabetes Association (ADA) do not limit the consumption of eggs or cholesterol, following the American Dietary Guidelines published in 2015 by the American Government [62]. Thus, changes are warranted to the Colombian Dietary Guidelines (published before the American Guidelines 2015–2020), which recommend only one egg per day in the diets of Colombians [63].

## 5. Conclusions

Eggs cannot be seen only as a source of cholesterol. Eggs also provide minerals, vitamins, antioxidants, and, importantly, a source of good-quality protein. Therefore, eggs represent an excellent option to be included as part of a balanced and healthy diet without increasing risk factors for CVD in our population, as was demonstrated in this study. The addition of Annatto did not modify any of the lipid biomarkers measured. Future studies should address the contribution of Annatto in modifying biomarkers of oxidative stress and inflammation when combined with eggs. In summary, this intervention may encourage the consumption of eggs as part of a healthy diet in this Colombian population, with the added advantage of their low cost, compared to other protein sources.

## 6. Limitations

Although it was possible to control the study for different factors such as age, sex, and BMI, it is important to consider that it was not possible to blind the intervention groups, and this could have implications for biases. Especially for the control group, the only natural food coloring available in the market was the same Annatto, limiting the possibility of masking the control group to simulate whole egg. In addition, the use of a parallel randomized clinical trial is a limitation, since it does not consider the individual characteristics of the volunteers, which play an important role in their metabolic and physiological responses.

## Figures and Tables

**Table 1:** Description of the study population according to the treatment groups.

			Egg Whites (Control)	Egg	Egg + Annatto	*p*-Value
			*n* (%)	*n* (%)	*n* (%)	
Sex	Women		24 (22.8)	23 (21.9)	22 (20.9)	0.88
	Men		11 (10.5)	12 (11.4)	13 (12.4)	
Age	Young adult (≥18 to ≤26 years)		12 (11.4)	12 (11.4)	11 (10.5)	0.958
	Adult (≥27 years)		23 (21.9)	23 (21.9)	24 (22.9)	
Socioeconomic level	1		2 (1.9)	0 (0.0)	0 (0.0)	0.251 *
	2		9 (8.6)	4 (3.8)	5 (4.8)	
	3		15 (14.2)	21 (20.0)	24 (22.9)	
	4		8 (7.6)	8 (7.6)	6 (5.7)	
	5		1 (0.9)	2 (1.9)	0 (0.0)	
Education level	High school		9 (8.6)	5 (4.8)	10 (9.5)	0.509
	Technical or technological		8 (7.6)	4 (3.8)	4 (3.8)	
	Professional		12 (12.5)	17 (17.7)	14 (14.5)	
	Postgraduate		6 (5.7)	9 (8.6)	7 (6.7)	
Body mass index (BMI)	Baseline	Normal (≥18.5 to ≤24.9 kg/m^2^)	24 (22.9)	19 (18.1)	21 (20.0)	0.467
		Overweight (≥25 kg/m^2^)	11 (10.4)	16 (15.2)	14 (13.3)	
	Week 8th	Normal (≥18.5 to ≤24.9 kg/m^2^)	25 (23.8)	20 (19.0)	21 (20.0)	0.424
		Overweight (≥25 kg/m^2^)	10 (9.5)	15 (14.3)	14 (13.3)	

* Fisher’s Exact Test.

**Table 2:** Effects on traditional cardiovascular risk markers in the study population according to the treatment.

	Egg Whites (Control)		Egg		Egg + Annatto		Value		
	Median (±SD)		Median (±SD)		Median (±SD)		p^1^	p^2^	p^3^
	Baseline	Week 8	Baseline	Week 8	Baseline	Week 8			
Total Cholesterol (mg/dL)	182 (32.2)	183 (36.9)	180 (37.2)	185 (37.4)	172 (32.4)	178 (36.2)	0.449	0.352	0.866
HDL Cholesterol (mg/dL)	52.1 (15)	51.1 (14.9)	53.7 (13.5)	52.8 (12)	50 (10)	50.2 (9.1)	0.697	0.441	0.855
LDL Cholesterol (mg/dL)	118 (30.8)	118 (28.9)	114 (32.4)	120 (33.3)	111 (33.1)	116 (36.6)	0.599	0.418	0.807
Triglycerides (mg/dL)	106 (53.6)	112 (69.5)	106 (40.8)	107 (39.4)	110 (60.8)	105 (57.7)	0.981	0.797	0.892
Glucose (mg/dL)	84.2 (7.8)	85.2 (10.6)	83.6 (6.9)	85.4 (5.4)	83.8 (5.8)	83.6 (5.9)	0.398	0.674	0.721

Repeated-measures ANOVA. p^1^: time, p^2^: treatment, p^3^: time x treatment.

**Table 3:** Effects on cardiovascular risk indexes and apolipoproteins in the study population according to treatment.

	Egg Whites (Control)		Egg		Egg + Annatto		Value		
	Median (±SD)		Median (±SD)		Median (±SD)		p^1^	p^2^	p^3^
	Baseline	Week 8	Baseline	Week 8	Baseline	Week 8			
TC/HDLc	3.67 (1.1)	3.82 (1.2)	3.49 (0.9)	3.61 (0.8)	3.57 (0.9)	3.64 (0.9)	0.363	0.719	0.981
LDL-C/HDL-C	2.45 (1.0)	2.5 (0.9)	2.24 (0.8)	2.36 (0.7)	2.35 (0.9)	2.4 (0.9)	0.428	0.623	0.959
Non-HDLC	129 (34.2)	132 (37.9)	126 (33.8)	132 (33.3)	122 (32.1)	128 (35.3)	0.333	0.585	0.922
TG/HDL-C	2.27 (1.6)	2.56 (2.2)	2.12 (1.0)	2.14 (0.9)	2.35 (1.4)	2.19 (1.4)	0.807	0.527	0.663
Apo B (mg/dL)	73.5 (19.5)	74.3 (22.5)	73.4 (23.3)	72.9 (24.5)	72.9 (24.5)	69.5 (22.9)	0.487	0.544	0.903
ApoA1 (mg/dL)	137 (21.9)	131 (23.3)	137 (26.1)	138 (24.1)	131 (17.7)	132 (18.2)	0.565	0.341	0.458
LDLc/ApoB	1.63 (0.22)	1.63 (0.23)	1.58 (0.20)	1.60 (0.19)	1.64 (0.22)	1.62 (0.16)	0.895	0.493	0.804
ApoB/ApoA1	0.54 (0.15)	0.59 (0.21)	0.55 (0.19)	0.56 (0.16)	0.54 (0.18)	0.56 (0.20)	0.345	0.840	0.949

Repeated-measures ANOVA. p^1^: time, p^2^: treatment, p^3^: time x treatment. Abbreviations: TC: total cholesterol; LDL-C: low-density lipoprotein cholesterol; HDL-C: high-density lipoprotein cholesterol; TG: triglycerides.

**Table 4:** Effects on lipoprotein subclasses’ concentrations and by size in the studied population according to treatment.

	Egg Whites		Egg		Egg + Annatto		Value		
	Median (±SD)		Median (±SD)		Median (±SD)		p^1^	p^2^	p^3^
	Baseline	Week 8	Baseline	Week 8	Baseline	Week 8			
Total Triglyceride-Rich Lipoprotein Particle (TRLP, nmol/L)	130 (44.2)	125 (43.9)	147 (47)	148 (38.1)	134 (45.9)	136 (49.9)	0.991	0.866	0.133
Very Large TRLP (nmol/L)	0.34 (0.5)	0.42 (0.5)	0.39 (0.5)	0.28 (0.3)	0.38 (0.4)	0.33 (0.3)	0.969	0.378	0.636
Large TRLP (nmol/L)	2.63 (1.5)	2.95 (2.0)	3.03 (1.6)	3.15 (2.2)	2.90 (1.9)	2.66 (2.0)	0.743	0.517	0.611
Medium TRLP (nmol/L)	13.5 (9.0)	15.4 (9.2)	12.2 (4.7)	13.2 (6.8)	12.1 (6.3)	11.6 (5.3)	0.374	0.511	0.670
Small TRLP (nmol/L)	54.8 (27.8)	53.7 (33.9)	56 (32.7)	63.9 (35.4)	54.9 (33.7)	65 (34)	0.295	0.571	0.483
Very Small TRLP (nmol/L)	59.2 (37.3)	66.9 (54.2)	71.0 (34.1)	57.3 (28.1)	56.1 (27.4)	49.2 (29.2)	0.264	0.176	0.363
Total LDL Particle (LDL-P, nmol/L)	1154 (290)	1217 (382)	1121 (317)	1157 (284)	1098 (371)	1104 (302)	0.447	0.318	0.876
Large LDL-P (nmol/L)	401 (190)	365 (182)	335 (129)	373 (112)	358 (175)	365 (182)	0.882	0.547	0.404
Medium LDL-P (nmol/L)	332 (1629	340 (181)	350 (267)	260 (162)	298 (167)	313 (180)	0.466	0.366	0.353
Small LDL-P (nmol/L)	361 (166)	477 (273)	377 (98)	468 (210)	386 (180)	449 (227)	0.959	0.235	0.898
Total Calibrated HDL Particle (cHDL-P, μmol/L)	20.3 (2.8)	19.3 (1.6)	20.5 (3.2)	20.3 (2.9)	19.9 (2.1)	19.8 (1.9)	0.326	0.365	0.422
Large cHDL-P (μmol/L)	2.52 (1.6)	1.81 (1.1)	1.83 (1.2)	2.12 (1.1)	2.13 (1.4)	2.16 (1.3)	0.975	0.873	0.155
Medium cHDL-P (μmol/L)	5.93 (2.6)	5.87 (1.9)	6.01 (1.7)	6.29 (2.0)	5.71 (1.7)	5.82 (1.7)	0.619	0.479	0.959
Small cHDL-P (μmol/L)	11.9 (3.3)	11.5 (2.9)	12 (3.3)	11.6 (3.0)	11.7 (2.1)	11.7 (2.3)	0.957	0.515	0.910

Repeated-measures ANOVA. p^1^: time, p^2^: treatment, p^3^: time x treatment.

## Data Availability

The data presented in this study are available on request from the corresponding author. The data are not publicly available as analyses are still underway for additional publications.
